# COX2 Enhances Neovascularization of Inflammatory Tenocytes Through the HIF-1α/VEGFA/PDGFB Pathway

**DOI:** 10.3389/fcell.2021.670406

**Published:** 2021-08-04

**Authors:** Bin Deng, Pu Xu, Bingyu Zhang, Qing Luo, Guanbin Song

**Affiliations:** ^1^Key Laboratory of Biorheological Science and Technology, Ministry of Education, College of Bioengineering, Chongqing University, Chongqing, China; ^2^Chongqing Engineering Research Center of Medical Electronics and Information Technology, College of Bioinformatics, Chongqing University of Posts and Telecommunications, Chongqing, China

**Keywords:** COX2, tendon, inflammatory injury, anti-angiogenesis, HIF-1α/VEGFA/PDGFB pathway

## Abstract

Tendon injuries are among the most challenging in orthopedics. During the early tendon repair, new blood vessel formation is necessary. However, excessive angiogenesis also exacerbates scar formation, leading to pain and dysfunction. A significantly worse outcome was associated with higher expression levels of hypoxia-inducible factor-1 alpha (HIF-1α), and its transcriptional targets vascular endothelial growth factor A (VEGFA) and platelet-derived growth factor B (PDGFB), but the underlying molecular mechanisms remain unclear. In this study, lipopolysaccharide (LPS) was used to induce an inflammatory response in tenocytes. LPS increased the tenocytes’ inflammatory factor COX2 expression and activated the HIF-1α/VEGFA/PDGFB pathway. Moreover, the conditioned medium from the tenocytes boosted rat aortic vascular endothelial cell (RAOEC) angiogenesis. Furthermore, Trichostatin A (TSA), an inhibitor of histone deacetylase, was used to treat inflammatory tenocytes. The expression levels of HIF-1α and its transcriptional targets VEGFA and PDGFB decreased, resulting in RAOEC angiogenesis inhibition. Finally, the dual-luciferase reporter gene assay and chromatin immunoprecipitation (ChIP) assay proved that the HIF-1α/PDGFB pathway played a more critical role in tenocyte angiogenesis than the HIF-1α/VEGFA pathway. TSA could alleviate angiogenesis mainly through epigenetic regulation of the HIF-1α/PDGFB pathway. Taken together, TSA might be a promising anti-angiogenesis drug for abnormal angiogenesis, which is induced by tendon injuries.

## Introduction

Tendons are responsible for transferring the mechanical loads generated by muscles to bones. However, excessive or inappropriate stretches often cause tendon injury. Due to its limited blood supply and slow cellular metabolism, tendon is difficult to repair following injury (Chen et al., 2011). How to cure the damaged tendon is still a troublesome problem.

Wound tissue repair occurs in tissues after injuries. The repair process represents a very complicated biological process, which needs multiple biological pathways to restore tissue function (Yoshikawa et al., 2006). During tissue repair, new blood vessel formation is necessary. However, the molecular mechanism of wound healing and tissue regeneration remains unclear (Gurtner et al., 2008). Abnormal proangiogenic factors also exacerbate scar formation, leading to pain and dysfunction (Korntner et al., 2019). Improper tissue healing can involve a delay or excessive recovery characterized by large amounts of extracellular matrix (ECM; Petersen et al., 2003). Generally, the early vasculature is not fully functional under the high proangiogenic pressure situation (Nagy et al., 2008). Therefore, the increase in angiogenesis should be regulated. Uncontrolled vessel growth may cause diseases, such as tendinitis, psoriasis, arthritis, and cancer (Li et al., 2019). Interestingly, recent studies have suggested that controlling the blood vessel density may lead to functional vasculature (Dallaudiere et al., 2013) and improve long-term healing outcomes (Mitchell et al., 2009). Moreover, anti-angiogenesis therapies will potentially reduce vascular regression and edema (Wang et al., 2015).

Vascular endothelial growth factor (VEGF) is a potent growth factor for angiogenesis. Although the VEGF family consists of seven members, the VEGF typically refers to the VEGFA isoform, which is the most studied member and a primary mediator of angiogenesis. VEGFA has been primarily identified for increasing vascular permeability (Zhou et al., 2011; Li et al., 2017). It was then identified for its ability to promote vascular endothelial cell growth and was named VEGFA. When cells secrete VEGFA, VEGFA interacts with cell surface receptors, such as VEGFR1 and VEGFR2, which are located on bone marrow-derived cells and vascular endothelial cells (Tempfer et al., 2018). VEGFR2 is responsible for the major angiogenic functions of VEGFA, whereas the role of VEGFR1 is not fully understood (Petersen et al., 2003). Some documents have reported that VEGFR1 can serve as a decoy receptor, preventing VEGFA from acting on VEGFR2 to activate downstream pathways. VEGFA and VEGFR2 are primary targets for antiangiogenic treatments (Petersen et al., 2003; Kuang et al., 2013).

However, the VEGF family is not the only vascular growth factor involved in tendon repair. Platelet-rich plasma (PRP) therapy is a new therapy for bone tissue injury in recent years. Its main action factor, platelet-derived growth factor B (PDGFB), is a peptide regulator that stimulates connective tissue and plays a role in forming fibroblasts and blood vessels (Tudoran et al., 2015). Current studies have reported that the expression of PDGFB is increased after surgery and during injury repair. Furthermore, PDGFRβ is the central receptor of PDGFB. Some studies have shown that PDGFB plays an essential role in injury repair (Kryza et al., 2014). Recently, hypoxia-inducible factor-1 alpha (HIF-1α) has been reported to bind the promoter region of *PDGFB*, suggesting that *HIF-1*α may target *PDGFB* (Liang et al., 2017).

HIF-1α, the master regulator of oxygen homeostasis, is enzymatically degraded under normoxia by propyl hydroxylases. VHL is part of an E3 ubiquitin ligase complex that recognizes HIF-1α subunits in normoxia (through hydroxylated proline residues), hence favoring ubiquitination and proteosome degradation of alpha subunits in normal oxygen conditions. HIF-1α has recently been characterized as an emerging master regulator in response to inflammation and the critical mediator of VEGFA activity (Ellis et al., 2009). The level of histone acetylations tightly controls the HIF-1α pathway (Ramzy et al., 2018). It is regulated by histone acetyltransferases (HATs) and histone deacetylases (HDACs; Bose et al., 2014). The role of HDACs in regulating angiogenesis was first investigated by Kim et al. They demonstrated that many cell lines exhibited the increased mRNA and protein expression of HDAC1, HDAC2, and HDAC3 under hypoxic conditions (Kim et al., 2001). In fact, p53 is the first non-histone protein that was found to be regulated by acetylation/deacetylation, and its carboxy-terminal lysine is the main target of acetylation regulation. P53 binds to p300, a transcriptional cofactor, which promotes the acetylation of p53 to enhance its transcriptional activity (Liu et al., 1999). P53 acetylation promotes HIF-1α degradation (Gu and Roeder, 1997). Overexpression of HDAC1 decreased p53 and protein von Hippel-Lindau (pVHL) expression. The decreased expression of the tumor suppressor gene p53 resulted in the overexpression of HIF-1α and VEGF. Some studies have indicated that histone deacetylase inhibitors (HDACIs) show excellent prospects for preventing angiogenesis (Li et al., 2015). For example, trichostatin A (TSA) is the most commonly used HDACI with intense activity, relatively low price, and clear structure (Bose et al., 2014). At present, there is little research on the molecular mechanism of angiogenesis in tendon injury. We hypothesize that tenocytes and cancer cells have a similar regulatory mechanism. As an effective component of the cell wall of Gram-negative bacteria, lipopolysaccharide (LPS) can effectively stimulate liver cancer cells to release inflammatory cytokines, inflammatory mediators, and adhesion molecules (such as IL-1β, IL-6, and TNF-α). Studies have indicated that after LPS stimulation of chondrocytes, the phosphorylation of MAPKs and the activation of NF-κB were induced, and the degradation products of proteoglycan and type II collagen, components of cartilage matrix, increased. Therefore, in this study, we used LPS to construct the inflammatory injury model of tenocytes. To further investigate the molecular mechanism of angiogenesis caused by tenocyte inflammation, and whether TSA can interfere with this process, we did a series of biochemical experiments. Accordingly, this study examined the TSA as a promising anti-angiogenesis drug for abnormal angiogenesis, which is induced by tendon injuries.

## Materials and Methods

### Ethical Approval

All animal procedures were approved by the Research Ethics Committee of the Army Medical University (SYXK-2012-0003) and in accordance with the US National Institutes of Health (NIH, 8th76 edition, 2011).

### Primary Culture of Rat Tenocytes and Aortic Endothelial Cells (RAOECs)

Sprague–Dawley rats (Laboratory Animal Center, the Third Military Medical University, China) weighing 100 g were used. Briefly, the cervical dislocation method was used to euthanize the rats. Then, in an ultra-clean workbench, the tendons of the extremities were isolated and cut to the size of 1.0–2.0 mm^3^; thereafter, the small tissue blocks were uniformly attached to the bottom of the 25 cm^2^ culture bottle. Afterward, 4 ml of DMEM (Gibco by Life Technologies, CA, United States, # 11965092) was supplemented with 10% FBS (HI-FBS, Sigma-Aldrich, MO, United States, # F4135). Antibiotic–Antimycotic (Gibco by Life Technologies, CA, United States, # 15240096) was added to the culture bottle. The culture bottle was placed upside down for 6 h to make the tissues stick to the bottom and invert the bottle. It can be carried out subculture after reaching 80% confluence or contact between tissue block and tissue block of the cell community. First, the tissue block was blown off and removed, and then washed with PBS. The tenocytes were digested with 0.25% trypsin with 0.02% EDTA (Gibco by Life Technologies, CA, United States, # 25200072), then divided into three bottles, marked as P1. They were all passed down in the same way. Tenocytes from passage 3 or 4 were used. Tenocytes were seeded at 2 × 10^5^ cells in 2 ml of DMEM in six-well plates and allowed to adhere 24 h before stimulation by LPS (0.25 μg/ml, Sigma, United States, #L2630-10MG). Then, tenocytes were incubated with LPS for 4 h or 12 h. RAOECs were donated from Xinqiao Hospital of the Third Military Medical University. The culture method was similar to that of tenocytes.

### Quantitative Real-Time PCR

About 10^5^ cells were collected by 1 ml of RNA lysis solution in each well. Total RNA was extracted from tendons using an RNA extraction kit (Bioteke, China, #RP1001) according to the manufacturer’s protocol. Following that, the concentration and the quality of total extracted RNA were measured by using a Nanodrop 2000 spectrophotometer (Thermo Fisher Scientific, United States), and then 1 μg of the extracted RNA was then reversely transcribed to cDNA using the PrimeScript RT reagent kit with a gDNA eraser (Takara, Japan, # RR037A). Quantitative PCR was performed using 2 × SYBR qPCR Mix (Bimake, United States, #B21202) on a Real-Time PCR system (Bio-Rad CFX Manager system, United States) following the manufacturer’s instruction. PCR reaction conditions were 30 s at 95°C, followed by 40 cycles at 95°C for 5 s and 30 s at 58°C. The specific primer sequences are listed in Supplementary Table 1 and the targeted gene expression levels were normalized to β*-Actin* as an internal standard.

### Western Blot Analysis

Cells were collected in 100 μl of lysis buffer (Beyotime, China, # P0013B) and thermal cracking for 5 min. The samples (30 μg/well) were separated by 12% SDS-PAGE gels and electrically transferred to PVDF membranes (Millipore Corp, MA, United States, # IPVH00010). After being blocked, the primary antibodies COX2 (Cell Signaling Technology, United States, #12282), HIF-1α (Cell Signaling Technology, United States, #14179), K-AC (Cell Signaling Technology, United States, #9441), VEGFR2 (Cell Signaling Technology, United States, #9698), PDGFRβ (Cell Signaling Technology, United States, #3161), and β-Actin (Cell Signaling Technology, United States, #8457) were added to membranes overnight at 4°C. The secondary antibodies (Beyotime, China, #A0208) were added to membranes for 40 min, and an ECL system was used to display bands (Millipore, CA, United States, # WBULS0500).

### Construction of pCDH-COX2 and psiCHECK2-pVEGFA Vectors

The ORF of rat COX2 (GenBank ID: 26198) was amplified by PCR with primer F: 5′-GAATTCATGCTC TTCCGAGCTGTGCTG-3′ and R: 5′-GCGGCCGCTTACAGC TCAGTTGAACGCCTT-3′. The reaction product was purified and digested with *Eco*RI and *Not*I and purified, followed by cloning into the PCDH-CMV-MCS-EF1-EGFP + Puro vector at the *Eco*RI and *Not*I sites. PCR amplified the promoter of rat VEGFA (GenBank ID: 26198) with primer F: 5′-CTCGAGGCTCAGCAGACCTGGGTGAG-3′ and R: 5′-GCG GCCGCCCGCGACTGGTCCGATGAA-3′. The reaction product was purified and digested with *Xho*I and *Not*I, again purified and cloned into the psiCHECK2 vector at the *Xho*I and *Not*I sites. The promoter of rat *PDGFB* (GenBank ID: 24628) was amplified by PCR with primer F: 5′-TCGTTTAAACCTAGAGCGGCCGCAAACACTTCCAGTCCC ATACC-3′ and R: 5′- ATTTTATTGCGGCCAGCGGCCGCTTC ACTCGCCCGCTAAA-3′. The reaction product was purified and digested with *Xho*I and *Not*I, again purified and cloned into the psiCHECK2 vector at the *Xho*I and *Not*I sites. The constructed expression plasmids were transfected into tenocytes using Lipofectamine 3000 (Invitrogen, United States, # L3000001).

### Tube Formation Assay

In order to evaluate the angiogenic activity of released factors from tenocytes subjected to LPS treatment, a tube formation assay was used modified from Arnaoutova and Kleinman (2010). The conditioned media of the LPS-treated tenocytes were harvested after 24 h. The flat bottom 96-well plates were coated with 100 μl of Matrigel (BD Matrigel Basement Membrane Matrix, United States, #356234). The RAOECs were resuspended in the conditioned media of tenocytes that had been LPS treated or untreated (controls) for 24 h. Then, 100 μl of the resuspended cells (10^4^ cells) were added to each well. After 6 h of incubation, the tubular networks that form in the Matrigel in each well were micrographed using a digital camera (AxioCam ICm 1, Zeiss, Germany) attached to an inverted microscope (Axio Observer.A1, Zeiss, Germany) with a 5 × objective lens. Then, the TIF format gray-scale images of biological and technical replicates were analyzed with AngioTool software in order to measure the total tube length per field.

### siRNA Transfection

Negative control siRNA and specific siRNAs were bought from Genepharma, China. The siRNA sequences are shown in Supplementary Table 2. After tenocyte or RAOEC seeding for 24 h, cells (10^5^ cells/well) were transfected with 2 μg of siRNA, using Lipofectamine 3000 (Invitrogen, United States, # L3000001) (5 μl/well) for 48 h, as suggested by the manufacturer’s instructions.

### Enzyme-Linked Immunosorbent Assay

Tenocytes, after transfected with pCDH-COX2 vector and treated with TSA, were cultured in DMEM supplemented with 10% fetal bovine serum for 48 h. The cell numbers were counted, the culture supernatants were collected, and the concentration was normalized to 10^5^ cells/ml. VEGFA and PDGFB in the supernatant (0.2 ml) were determined. The concentrations of VEGFA and PDGFB were detected with rat ELISA kits (Neobioscience, China, #GTX00343-pro) and PDGFB (Neobioscience, China, #NOV-BG-RAT11754-96T). The readings were taken using a Bio-Rad enzyme-linked immunosorbent assay reader (Bio-Rad, United States, # 1681130A).

### Luciferase Reporter Assay

The psiCHECK2-pVEGFA reporter vector was co-transfected with the pCDH-COX2 vector into tenocytes. After 24 h culture, the cell culture medium was replaced with the medium including or excluding the TSA for 24 h, then the cell lysates were collected, and they were analyzed using the Beyotime Dual-Luciferase Reporter Assay System (Beyotime, China, #RG027).

### Immunoprecipitation Assay

Tenocytes were seeded in a 10-cm dish (5 × 10^6^ cells per dish) and transfected with a 16-μg pCDH-COX2 vector using Lipofectamine 3000 (Invitrogen, United States, # L3000001) (40 μl/dish) for 48 h, as suggested by the manufacturer’s instructions. Total protein samples were collected and lysed with RIPA lysis buffer (Beyotime, China, #P0013D) with protease inhibitor cocktail (Bimake, United States, #B14001) and cleared by centrifugation at 12,000 rpm. The supernatants were incubated at 4°C for 4 h with protein A + G beads (Beyotime, China, #P2012) in the presence of indicated antibodies COX2 (Cell Signaling Technology, United States, #12282), HIF-1α (Cell Signaling Technology, United States, #14179), p53 (Cell Signaling Technology, United States, #32532), or IgG (Beyotime, China, #A7016) as control. The immunoprecipitated proteins were subjected to immunoblotting as described in Western blotting.

### ChIP Assay

Tenocytes were seeded in a 10-cm dish (5 × 10^6^ cells per dish) and transfected with a 16-μg pCDH-COX2 vector using Lipofectamine 3000 (Invitrogen, United States, # L3000001) (40 μl/dish) for 48 h, as suggested by the manufacturer’s instructions. Chromatin immunoprecipitation (ChIP) assays were performed using the ChIP Assay Kit (Beyotime, China, #P2078). Briefly, the cells were fixed with 1% formaldehyde for 10 min, and the fixation reaction was quenched with glycine to a final concentration of 125 mM. The cells were lysed and sonicated until the desired lengths were achieved (100–250 bp). Then, anti-HIF-1α (5 μg) (Cell Signaling Technology, United States, #14179) and control IgG (Beyotime, China, #A7016) were used for immunoprecipitation. After elution of DNA from precipitated immunocomplexes, PCR or Q-PCR was performed with specific primers (Supplementary Table 1).

### Statistical Analysis

The data are shown as the mean ± standard deviation (SD), and the results were statistically analyzed using the Student’s *t*-test and analysis of variance. *p*-value <0.05 was considered to be statistically significant.

## Results

### The Construction of the Tenocyte Inflammation Injury Model

**FIGURE 1:**
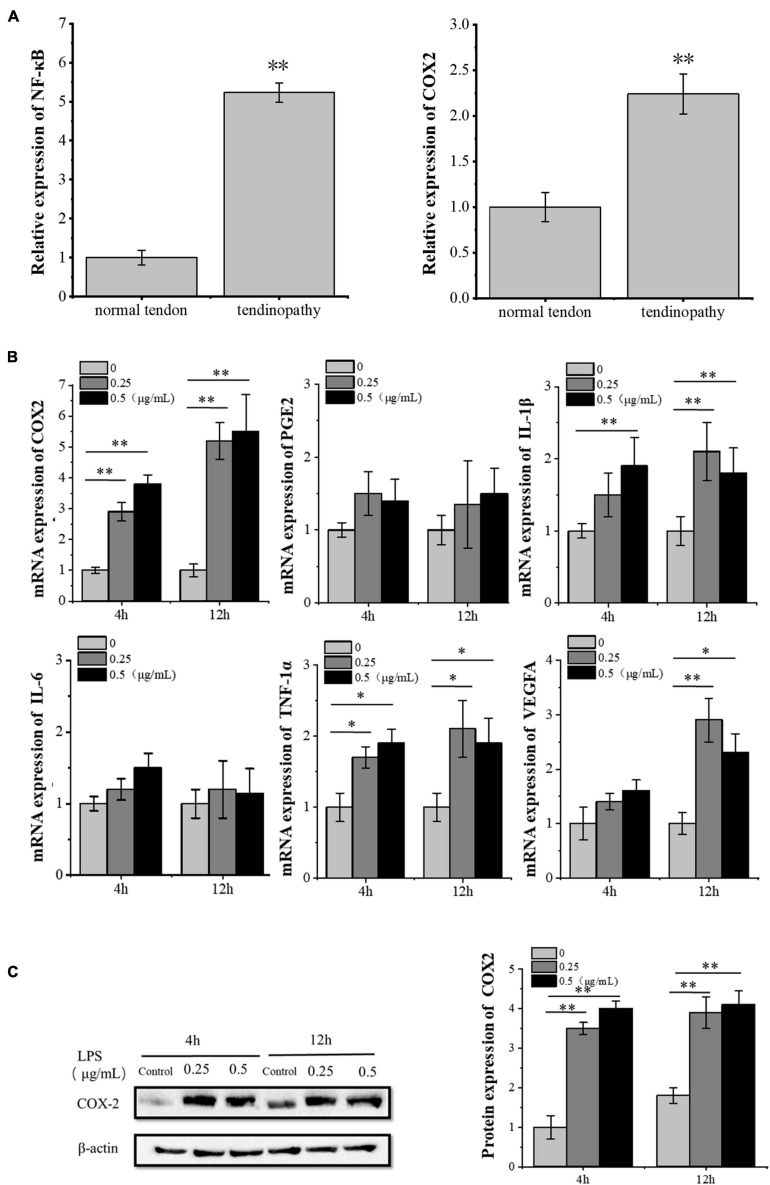
LPS induces inflammation in tenocytes. **(A)** The significant activation of the NF-κB pathway and the significantly high expression of inflammatory factor *COX2* in tendon patients were detected, based on the analysis of the expression profile of 23 tendon patients and 23 normal people with NCBI GEO GSE26051. **(B)** Q-PCR was performed to detect the mRNA expression levels of inflammatory factors downstream of the NF-κB pathway and *VEGFA* in tenocytes after LPS treatment. **(C)** Western blot was performed to detect the protein expression level of COX2. The data were presented as the means ± SD; *n* = 3; ^∗^*p* < 0.05, ^∗∗^*p* < 0.01. Microarray analysis, qPCR, and Western blot were performed using tenocytes. LPS concentration was 2.5 μg/ml.

We analyzed the data from Gene Expression Omnibus (GEO) datasets^1^ and found the significant activation of the NF-κB pathway in tendinopathy samples as compared to normal tendon tissue. Furthermore, we confirmed the significantly high expression of inflammatory factor *PTGS2* (*COX2*) in tendon patient samples (Figure 1A). We also confirmed the increase of the *COX2* mRNA and protein expression in LPS-treated tenocytes. In contrast, other inflammatory factors downstream of the NF-κB pathway did not increase significantly (Figures 1B,C). Besides, the *VEGFA* mRNA expression increased significantly after 12 h LPS-treated tenocytes. Besides, the concentration of 0.25 μg/ml LPS significantly increased *VEGFA* mRNA expression level compared to 0.5 μg/ml LPS (Figure 1B). So, for the following experiments, we chose 0.25 μg/ml LPS treatment for 12 h as the condition to induce the inflammation of tenocytes.

### The Effects of Inflammatory Injury of Tenocytes on Angiogenesis

**FIGURE 2:**
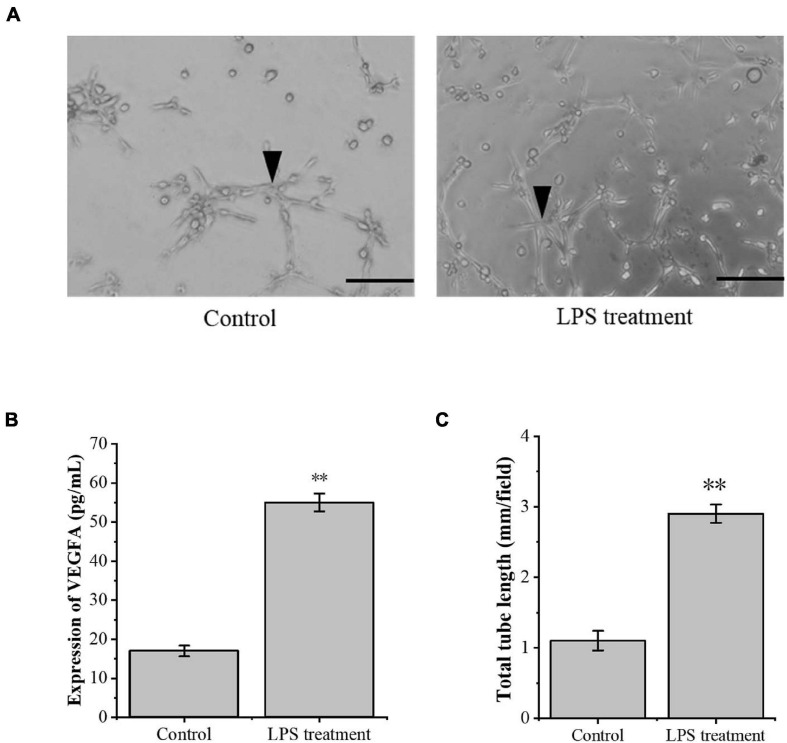
The inflammation tenocytes induced the protein expression level of VEGFA and tube formation of RAOECs. **(A,C)** A three-dimensional Matrigel assay was used to assess the tube formation of RAOECs. **(B)** ELISA was conducted to detect the protein expression level of VEGFA. The data were presented as the means ± SD; *n* = 3; ^∗∗^*p* < 0.01; scale bar: 50 μm; the triangles represent the position of the tubes. ELISA was performed using tenocytes, and the three-dimensional Matrigel assay was performed using RAOECs. LPS concentration was 2.5 μg/ml.

The model of tenocyte inflammation was established according to the above conditions. To study the effect of inflammatory tenocytes on angiogenesis, we collected the supernatant of the medium for ELISA detection. The results showed that the LPS-treated tenocytes still secreted significantly more VEGFA than the control group at 24 h (Figure 2B). RAOECs treated with a conditioned medium from the above LPS-treated tenocytes significantly enhanced the tubular-forming ability (Figures 2A,C). This experiment demonstrated a significant increase in the angiogenesis of inflammatory tenocytes.

### The Transcription Factor HIF-1α Is Involved in the Molecular Mechanism of Angiogenesis in Tenocyte Inflammation

**FIGURE 3:**
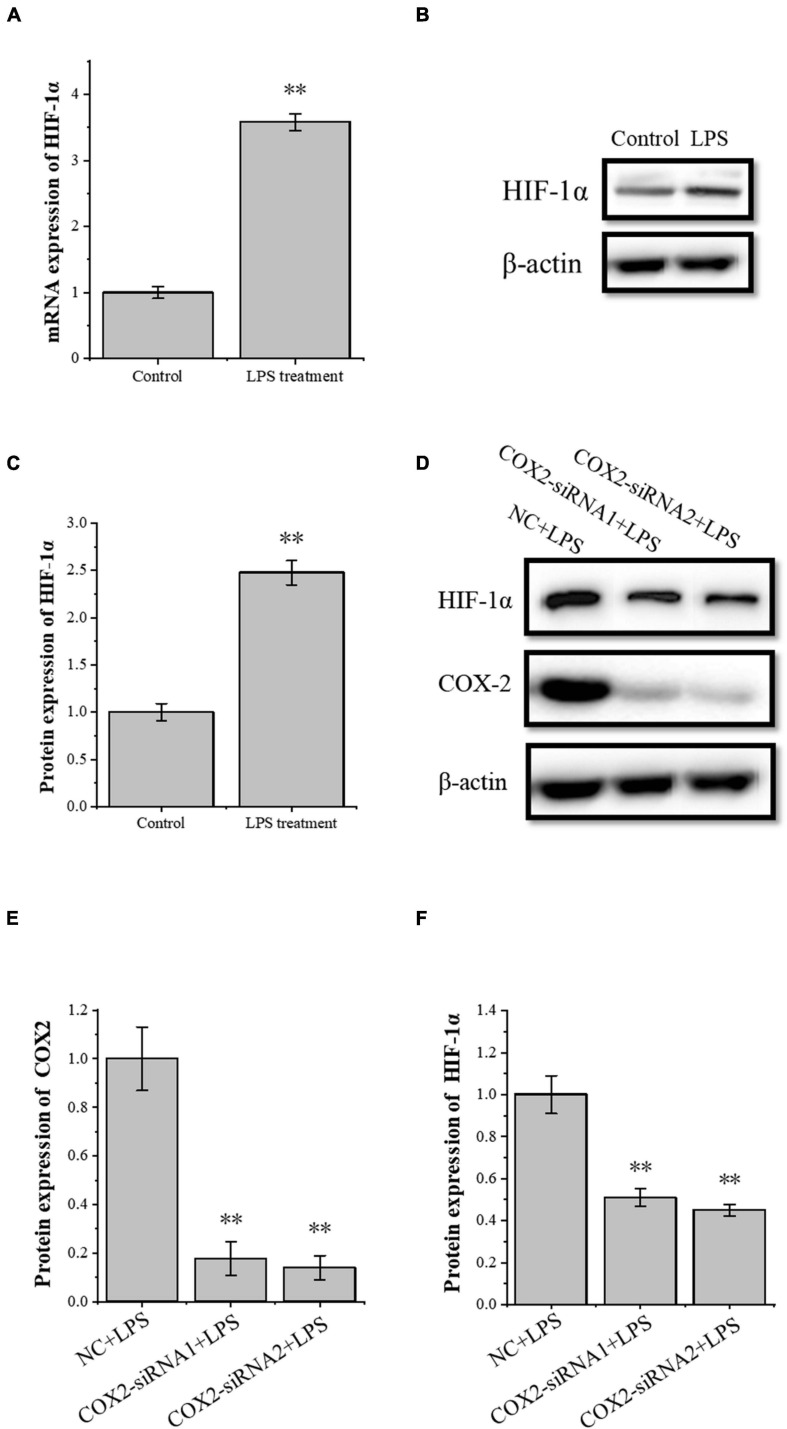
The relationship of the expression levels of inflammatory factor COX2 and transcription factor HIF-1α. **(A)** Q-PCR was performed to detect the mRNA expression levels of HIF-1α in tenocytes’ inflammation. **(B,C)** Western blot was performed to detect the protein expression level of HIF-1α in tenocytes’ inflammation. **(D–F)** Western blot was performed to detect the protein expression level of HIF-1α in tenocytes’ inflammation with COX2-siRNA transfection. The data were presented as the means ± SD; *n* = 3; ^∗∗^*p* < 0.01. Q-PCR and Western blot were performed using tenocytes. LPS concentration was 2.5 μg/ml.

**FIGURE 4:**
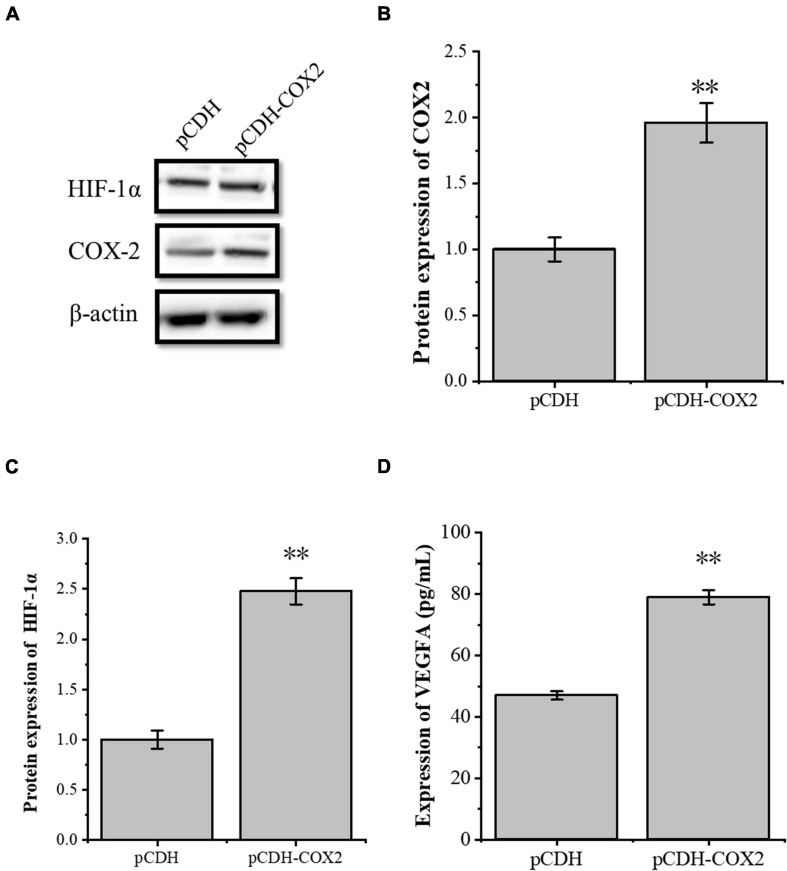
The overexpression of the inflammatory cytokines COX2 is associated with an increased expression level of transcription factors HIF-1α and VEGFA. **(A–C)** Western blot was conducted to detect the protein expression level of HIF-1α in tenocytes with pCDH-COX2 transfection. **(D)** ELISA was conducted to detect the protein expression level of VEGFA in tenocytes with pCDH-COX2 transfection. The data were presented as the means ± SD; *n* = 3; ^∗∗^*p* < 0.01. Western blot and ELISA were performed using tenocytes.

The transcription factor HIF-1α is involved in the angiogenesis of a lot of tumors. To investigate the role of HIF-1α in the angiogenesis of inflammatory tenocytes, q-PCR and Western blot were performed to detect the *VEGFA* key transcription factor *HIF-1*α mRNA and protein expression level, respectively. The HIF-1α expression of the LPS-treated group was significantly higher than the control group (Figures 3A–C). Tenocytes were transfected with COX2-siRNA for 24 h, followed by LPS treatment for 12 h. It showed that the siRNA significantly interfered with the expression of COX2 caused by LPS treatment. Moreover, the expression level of LPS-induced HIF-1α was also downregulated, suggesting that the expression levels of HIF-1α and VEGFA in tenocytes were related to COX2 (Figures 3D–F). The COX2 ORF area was constructed into eukaryotic expression vector pCDH, building a restructuring pCDH-COX2 plasmid. Then, it was transfected into the tenocytes. After 48 h, the tenocytes of pCDH-COX2 plasmid transfection highly expressed COX2, and the expression levels of HIF-1α and VEGFA also increased (Figure 4). This study showed that LPS-induced angiogenesis of inflammatory tenocytes might be related to the high expression levels of COX2, HIF-1α, and VEGFA.

### The Inflammatory Factor COX2 Plays a Role Through the VEGFA Pathway

**FIGURE 5:**
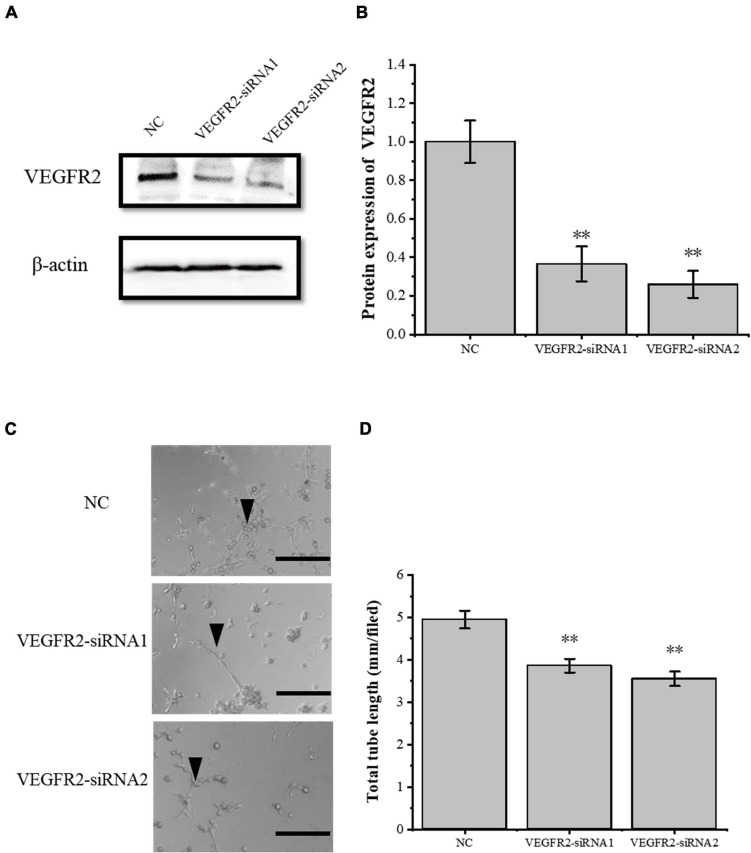
Knockdown of VEGFR2 inhibited the tube-forming ability of RAOECs. **(A,B)** Western blot was conducted to detect the protein expression level of VEGFR2 in RAOECs with VEGFR2-siRNA transfection. **(C,D)** A three-dimensional Matrigel assay was used to assess the tube formation of RAOECs with VEGFR2-siRNA transfection. The data were presented as the means ± SD; *n* = 3; ^∗∗^*p* < 0.01; scale bar: 50 μm; the triangles represent the position of the tubes. Western blot and the three-dimensional Matrigel assay were performed using RAOECs.

To verify the role of the VEGFA signaling pathway in the tube-forming ability of RAOECs, we used siRNA interference assay to knock down VEGFR2, which is the primary VEGFA receptor. The siRNA-induced VEGFR2 depletion was confirmed by Western blot (Figures 5A,B), and then RAOECs were treated with the conditioned medium from COX2-overexpressing tenocytes. The results showed that the tube-forming ability of RAOECs was inhibited (Figures 5C,D), demonstrating that the inflammatory factor COX2 plays a role through the VEGFA pathway.

### TSA Inhibits HIF-1α Expression and Its Downstream Target Gene VEGFA, Thereby Inhibiting Angiogenesis

**FIGURE 6:**
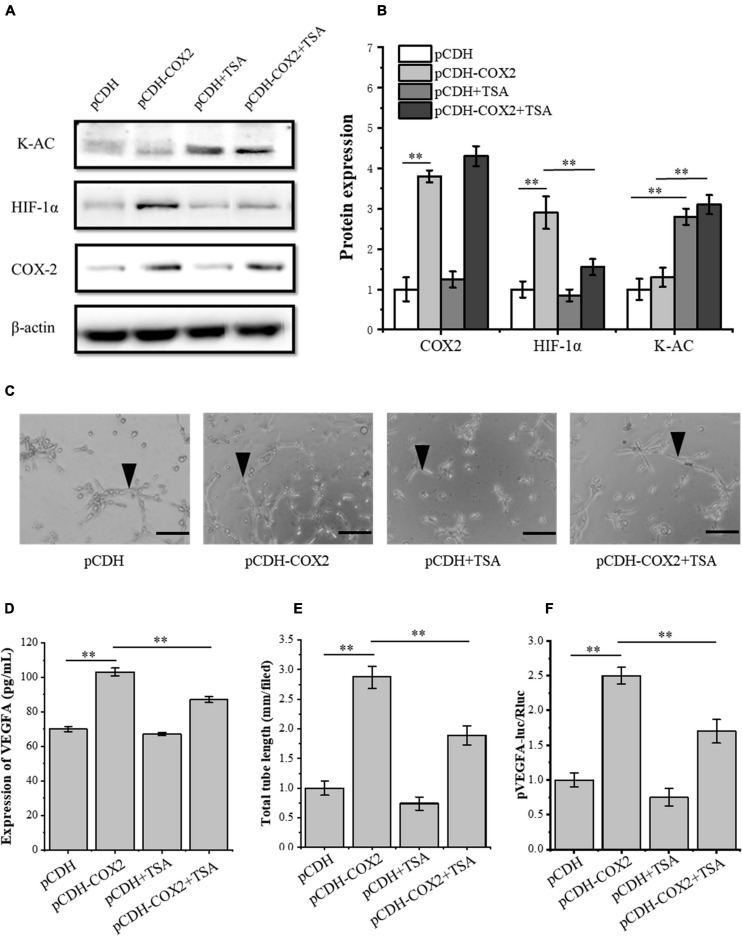
TSA affected the expression of HIF-1α and VEGFA in COX2 overexpressed tenocytes, the tube-forming ability of RAOECs, and the promoter activity of *VEGFA*. **(A,B)** Western blot was conducted to detect the protein expression level of K-AC, HIF-1α, and COX2. **(D)** ELISA was conducted to detect the protein expression level of VEGFA. **(C,E)** A three-dimensional Matrigel assay was performed to assess the tube formation of RAOECs. **(F)** The double luciferase reporter assay demonstrated the promoter activity of VEGFA. The data were presented as the means ± SD; *n* = 3; ^∗∗^*p* < 0.01; scale bar: 50 μm; the triangles represent the position of the tubes. Western blot and the double luciferase reporter assay were performed using tenocytes. The three-dimensional Matrigel assay was performed using RAOECs. TSA concentration was 500 nM.

To further study the effect of TSA on angiogenesis of inflammatory tenocytes, the pCDH-COX2 plasmid and empty PCDH controlled plasmid were transfected into the tenocytes and then replaced for medium treatment, including or excluding TSA after 24 h. Western blot was used for protein detection and the supernatant fluid was used for ELISA experiments. The result showed that the tenocytes of transfection of pCDH-COX2 plasmid increased COX2, HIF-1α, and VEGFA expression levels. The TSA improved the tenocyte acetylation level and inhibited the expression levels of HIF-1α and VEGFA (Figures 6A,B,D). The conditioned medium from COX2-overexpressing tenocytes significantly enhanced the tube-forming ability of RAOECs, which TSA inhibited (Figures 6C,E). The double luciferase reporter assay demonstrated that the *VEGFA* promoter activity was significantly enhanced by COX2 overexpression. In contrast, it was inhibited by TSA (Figure 6F). This experiment indicated that TSA inhibited angiogenesis of inflammatory tenocytes *via* the HIF-1α-VEGFA signaling pathway.

### TSA Inhibits HIF-1α Expression and Transcriptional Activity, Thereby Inhibiting PDGFB Expression and Angiogenesis

**FIGURE 7:**
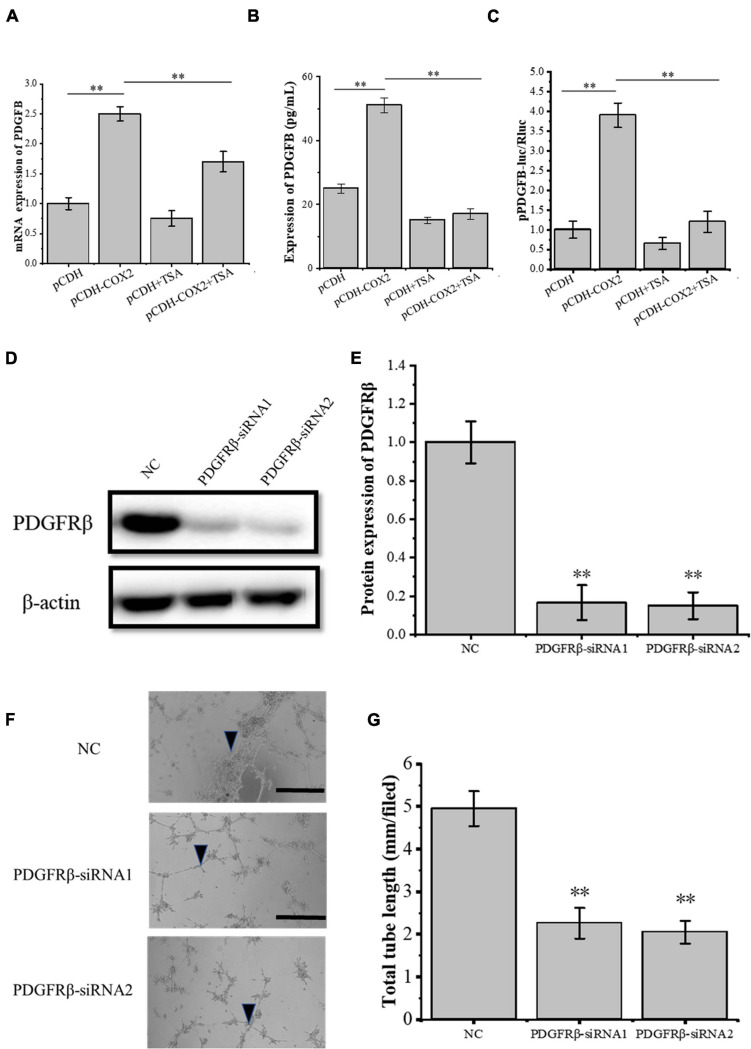
The expression of PDGFB in COX2 overexpressed tenocytes affects angiogenesis. **(A)** Q-PCR was performed to detect the mRNA expression level of *PDGFB*. **(B)** ELISA was conducted to detect the protein expression level of PDGFB. **(C)** The double luciferase reporter assay demonstrated the promoter activity of *PDGFB*. **(D,E)** Western blot was conducted to detect the protein expression level of PDGFRβ in RAOECs with PDGFRβ-siRNA transfection. **(F,G)** A three-dimensional Matrigel assay was performed to assess the tube formation of RAOECs with PDGFRβ-siRNA transfection. The data were presented as the means ± SD; *n* = 3; ^∗∗^*p* < 0.01; scale bar: 50 μm; the triangles represent the position of the tubes. Q-PCR, Western blot, ELISA, and the double luciferase reporter assay were performed using tenocytes. The three-dimensional Matrigel assay was performed using RAOECs. TSA concentration was 500 nM.

Through siRNA interference with VEGFR2, we found that VEGFR2 knockdown did not completely inhibit the angiogenesis of RAOECs, so there may be other signaling pathways to induce the tube-forming ability of RAOECs. In order to study this, the pCDH-COX2 plasmid and empty PCDH controlled plasmid were transfected into the tenocytes and then replaced for medium treatment including or excluding TSA after 24 h. Western blot was used for protein detection and the supernatant fluid was used for ELISA experiments. The result showed that transfection of pCDH-COX2 plasmid for tenocytes increased PDGFB expression level, while TSA inhibited it (Figures 7A,B). The double luciferase reporter assay demonstrated that the *PDGFB* promoter activity was significantly enhanced by COX2 overexpression. In contrast, it was inhibited by TSA (Figure 7C). The siRNA-induced PDGFRβ depletion was confirmed by Western blot (Figures 7D,E). The RAOECs were treated with the conditioned medium from COX2-overexpressing tenocytes. The results showed that the tube-forming ability of RAOECs was inhibited (Figures 7F,G), demonstrating that COX2 indeed plays a role through the PDGFB pathway. Moreover, the effect of PDGFB pathway inhibition on suppressing the tube-forming ability of RAOECs was stronger than the VEGFA pathway inhibition.

### The Crosstalk Between VEGFA and PDGFB Pathway During Angiogenesis

**FIGURE 8:**
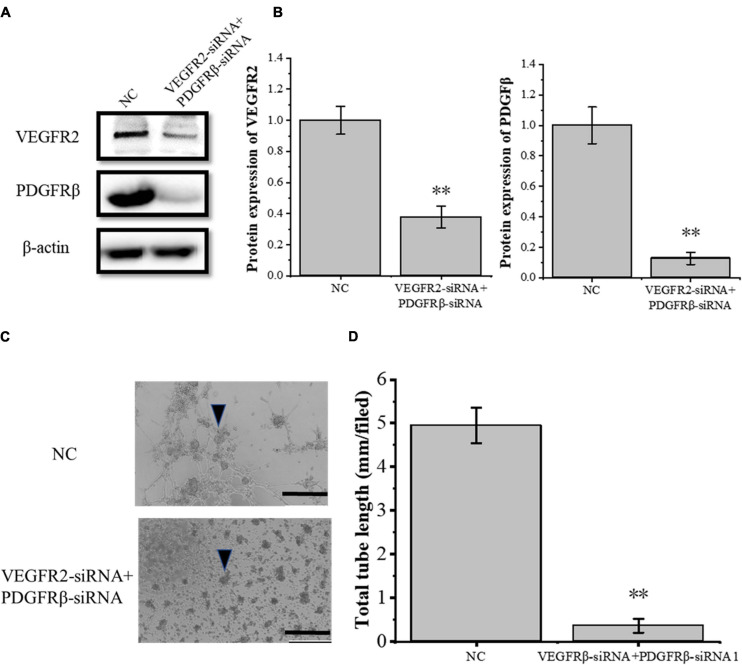
Knockdown of VEGFR2 and PDGFRβ inhibited the tube-forming ability of RAOECs of the conditioned medium from COX2-overexpressing tenocytes. **(A,B)** Western blot was performed to detect the protein expression level of VEGFR2 and PDGFRβ in RAOECs with VEGFR2-siRNA and PDGFRβ-siRNA transfection. **(C,D)** A three-dimensional Matrigel assay was conducted to assess the tube formation of RAOECs with VEGFR2-siRNA and PDGFRβ-siRNA transfection. The data were presented as the means ± SD; *n* = 3; ^∗∗^*p* < 0.01; scale bar: 50 μm; the triangles represent the position of the tubes. Western blot was performed using tenocytes. The three-dimensional Matrigel assay was performed using RAOECs.

To study the crosstalk between the VEGFA and the PDGFB pathway, the siRNA-induced VEGFR2 and PDGFRβ depletions were confirmed by Western blot (Figures 8A,B) and then the RAOECs were treated with the conditioned medium from COX2-overexpressing tenocytes. The results showed that the tube-forming ability of RAOECs was inhibited (Figures 8C,D). Moreover, The VEGFR2 and PDGFRβ knockdown resulted in a more significant decrease in the tube-forming ability of RAOECs than the VEGFR2 knockdown alone. It further demonstrated that the inflammatory factor COX2 plays a role through both VEGFA and PDGFB pathways.

### The Interactions of p53- and HIF-1α-Associated Proteins in COX2-Overexpressing Tenocytes

**FIGURE 9:**
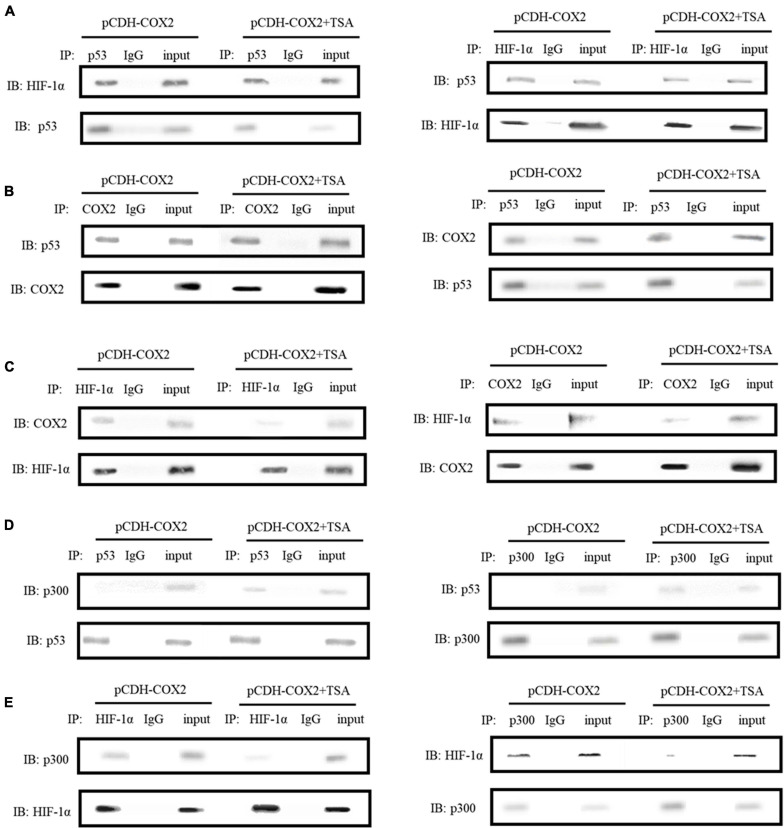
Immunoprecipitations were carried out to detect the interactions between COX2, p53, and HIF-1α, as well as p53, p300, and HIF-1α. **(A)** The interaction between HIF-1α and p53. **(B)** The interaction between COX2 and p53. **(C)** The interaction between COX2 and HIF-1α. **(D)** The interaction between p53 and p300. **(E)** The interaction between HIF-1α and p300. Immunoprecipitations were performed using tenocytes. TSA concentration was 500 nM.

In order to further study the molecular mechanism of COX2 promoting angiogenesis of inflammatory tenocytes, the interactions of COX2, p53, and HIF-1α, as well as p53, p300, and HIF-1α were detected using protein immunoprecipitation. The results showed direct interactions between COX2, p53, and HIF-1α, as well as p300 and HIF-1α in COX2-overexpressing tenocytes (Figures 9A,B,C,E). The same effects were observed during the addition of TSA, except that the interaction between COX2 and HIF-1α, as well as that between p300 and HIF-1α, decreased (Figures 9C,E). However, we did not observe the interaction between p53 and p300 in COX2-overexpressing tenocytes; it could be observed during the addition of TSA (Figure 9D). Thus, this study demonstrated that TSA inhibited the binding of COX2 and p300 to HIF-1α and promoted the binding of p300 to p53, which might be responsible for the inhibition of angiogenesis.

### TSA Inhibits HIF-1α Binding to VEGFA and PDGFB Promoters

**FIGURE 10:**
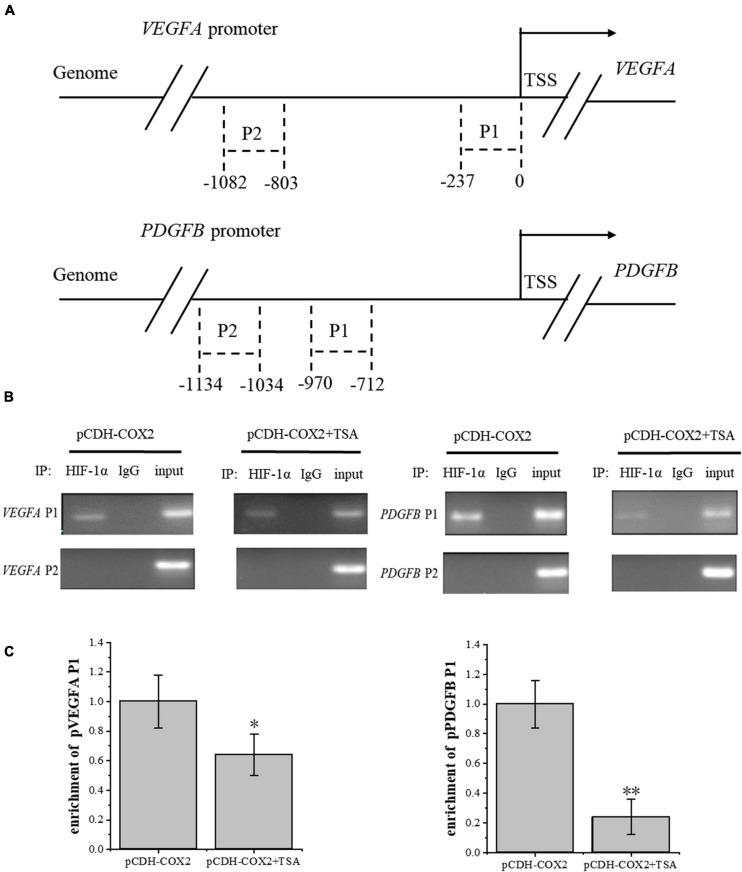
ChIP assay was conducted to detect the HIF-1α enrichment on *VEGFA* and *PDGFB* promoters. **(A)** Schematic diagram of the upstream promoter regions of the *VEGFA* and *PDGFB* genes. **(B)** Nucleic acid electrophoresis of ChIP-PCR products. **(C)** ChIP-qPCR detected the HIF-1α enrichment on *VEGFA* and *PDGFB* promoters. The data were presented as the means ± SD; *n* = 3; *p < 0.05, **p < 0.01. ChIP assay was performed using tenocytes. TSA concentration was 500 nM.

To investigate the effect of TSA on the HIF-1α regulation of downstream angiogenic genes, we used the AnimalTFDB database from Huazhong University of Science and Technology to predict the binding sites of transcription factor HIF-1α to *VEGFA* and *PDGFB* promoters in rats. Then, we designed primers before and after the binding sites. Moreover, PCR detection to DNA fragments extracted from ChIP was performed. The results showed that HIF-1α was significantly bound at the P1 sites of both gene promoters, but not at P2 sites, while TSA inhibited the HIF-1α binding in COX2-overexpressing tenocytes (Figures 10A,B). Furthermore, the inhibition of HIF-1α binding to the *PDGFB* promoter was higher than that of *VEGFA* (Figure 10C). This might be the main reason for TSA to inhibit angiogenesis through HIF-1α.

## Discussion

Some studies have reported that angiogenesis inhibitors also repress the expression level of HIF-1α, which is the critical mediator of VEGFA (Fang et al., 2004). The HIF transcription factor family consists of constitutively expressed β subunits such as HIF-1β, the expression level of which is kept constant. However, HIF-1α is sensitively regulated by oxygen levels. It is expressed in all tissues and plays a key role (Lv et al., 2017). During normoxia, HIF-1α combines with von Hippel-Lindau (VHL) protein. VHL is part of an E3 ubiquitin ligase complex that recognizes HIF-1α subunits in normoxia (through hydroxylated proline residues), hence favoring ubiquitination and proteosome degradation of alpha subunits in normal oxygen conditions (Lv et al., 2017). In contrast, hypoxia stabilizes HIF-1α, which can then escape from pVHL-mediated degradation and bind to p300 and CBP (Li et al., 2017). This complex then translocates to the nucleus from the cytoplasm and forms a heterodimer with HIF-1β to initiate the transcription of downstream targets such as VEGFA (Chen et al., 2015). The expression of inflammation-induced HIF-1α enhances cell metabolic activity and raises the oxygen consumption rate (Wu et al., 2017). Inflammation factors cause vasoconstriction and reduce oxygen in the inflammation area. Finally, a hypoxic microenvironment is formed. Some studies have shown that LPS and other inflammatory factors can activate the expression level of HIF-1α, indicating the close relationship between HIF and inflammatory processes (Kim et al., 2007). Our results suggested that, in tenocytes, LPS could mainly induce the expression level of COX2 and activate HIF-1α. Our lab used a cell stretch loading device. However, there was no significant effect on COX2 and other inflammatory factor expression levels. We promoted the mRNA expression level of *COX-2* in rat tenocytes by TNF-α, but the expression level was not significantly higher than LPS, and LPS was more economical. However, these results need to be verified with *in vivo* experiments, and further mechanisms still need intensive study. Consistent with the central role of HIF in the hypoxic response, targeted inactivation of HIF-1α in the mouse leads to embryonic lethality due to abnormal vascular development. The defects in vasculature have been observed in the yolk sac as well as in the developing embryonic tissue and are associated with severe hypoxia in the HIF-1α^–/–^ embryos. Mice with heterozygous defects of HIF-1α have a reduced protective effect of hypoxic preconditioning in a model of cardiac ischemia and a dramatic effect on carotid body neural activity and ventilatory adaptation to chronic hypoxia (Weidemann and Johnson, 2008). So, at what stage of tendon injury repair should TSA treatment be applied is a complicated question.

Angiogenic growth factors, such as VEGFA and PDGFB, express an increase in injured tissue than healthy tissue. Transcriptional activation is caused by HIF-1α binding to its target DNA site HRE (5-ACGT-3), promoting the overexpression of *VEGFA* and *PDGFB* (Kaelin and Ratcliffe, 2008). Some studies have shown that VEGFA is the most major vascular growth factor in tumor angiogenesis (Beck et al., 2011). However, in this study, from the effects of the corresponding receptors’ knockdown on the tube-forming ability of RAOECs (Figures 6–8), the dual-luciferase reporter assay showing HIF-1α activation activity (Figures 6F, 7C), and the ChIP assay demonstrating the binding ability of HIF-1α to the corresponding promoters (Figure 10), PDGFB should play a more important role in tendon angiogenesis than VEGFA. TSA inhibited tendon angiogenesis mainly through the PDGFB pathway.

**FIGURE 11:**
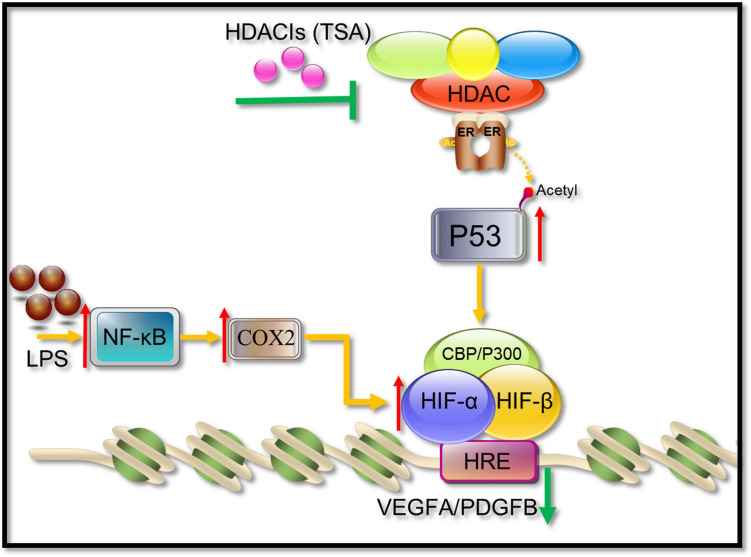
A possible pathway showing HDACIs (TSA) may suppress angiogenesis. HIF-1α binds to HIF-1β and recruits CBP/p300, resulting in the increase in transcriptional levels of downstream angiogenic growth genes such as *VEGFA* and *PDGFB*. TSA reverses the activity of HDACs, leading to an increasing level of acetylation in tenocytes. The acetylated p53 suppressed the HIF-1α binding activities to *VEGFA* and *PDGFB* promoters.

HIF-1α and its activation activity are vital mediators of VEGFA and PDGFB. HDACI TSA is involved in various mechanisms suppressing HIF-1α, such as (1) regulation through the TGF-β pathway and the induction of the expression of non-coding RNAs targeting HIF-1α (Hsieh et al., 2015; Liu et al., 2015); (2) HDACIs can acetylate many non-histone proteins, including many transcription factors and molecular chaperones, which can be activated or inhibited by acetylation to produce many different effects (Ellis et al., 2009). For example, p53 is the first non-histone protein regulated by acetylation/deacetylation, and its carboxy-terminal lysine is the main target of acetylation regulation. The p53 acetylation inhibits HIF-1α function (Deng et al., 2020; Figure 11). In this study, in COX2-overexpressing tenocytes, COX2 could promote the ability of HIF-1α to bind to downstream *VEGFA* and *PDGFB* promoters, and p53 could also bind to the COX2–HIF-1α complex. During TSA treatment, p53 bound to p300, which promoted the acetylation of p53 to enhance its transcriptional activity. Acetylated p53 might inhibit HIF-1α function by preventing the binding of the COX2, p300, and HIF-1α complex (Figure 9). This could be due to the fact that the acetylation of p53 might affect the binding between other proteins (Semenza, 2014). TSA is the most commonly used HDACI with intense activity, a relatively low price, and a clear structure. Some studies indicated that TSA (500 nM) decreased HDAC class I/II activity and that there was no obvious cytotoxicity (Azechi et al., 2013), and our study confirmed it. In addition, benzamide inhibitors are a new class of HDACIs. Compared with TSA, these HDACIs have stronger HDAC1/2 selectivity, lower toxicity, and better tolerability due to the unique N-(2-aminophenyl) benzamide pharmacodynamic group. At present, phenylpropanamide HDACIs in clinical trials mainly include Entinostat (MS-275), MGCD0103, and CS-055, among others (Ryu et al., 2019). Such compounds may also be better at anti-angiogenesis.

Overall, during the tendon repair, new blood vessels are necessary (Kang et al., 2012). However, abnormal proangiogenic factors also exacerbate scar formation, resulting in pain and dysfunction (Cook and Figg, 2010). Recent studies have suggested that controlling the blood vessels leads to functional vasculature and improves long-term healing outcomes (Sahin et al., 2012). Our results first indicated that TSA could alleviate angiogenesis mainly through epigenetic regulation of the HIF-1α/PDGFB pathway. Taken together, TSA can be a promising anti-angiogenesis drug for abnormal angiogenesis, which is induced by tendon injuries.

## Data Availability Statement

Publicly available datasets were analyzed in this study. This data can be found here: http://www.ncbi.nlm.nih.gov/geo/query/acc.cgi?acc=GSE26051.

## Ethics Statement

The animal study was reviewed and approved by the Research Ethics Committee of the Army Medical University [SYXK-2012-0003].

## Author Contributions

BD performed the experiments and wrote the manuscript. PX and BZ performed data processing and statistical analysis. GS and QL conceived and designed the experiments and revised the manuscript. All authors have read and agreed to the published version of the manuscript.

## Conflict of Interest

The authors declare that the research was conducted in the absence of any commercial or financial relationships that could be construed as a potential conflict of interest.

## Publisher’s Note

All claims expressed in this article are solely those of the authors and do not necessarily represent those of their affiliated organizations, or those of the publisher, the editors and the reviewers. Any product that may be evaluated in this article, or claim that may be made by its manufacturer, is not guaranteed or endorsed by the publisher.

## Footnotes

1
http://www.ncbi.nlm.nih.gov/geo/query/acc.cgi?acc=GSE26051
