# Supplementary material for: COX2 Enhances Neovascularization of Inflammatory Tenocytes Through the HIF-1α/VEGFA/PDGFB Pathway
Source: Front Cell Dev Biol. 2021 Aug 4;9:670406. doi: 10.3389/fcell.2021.670406 (PMC8371918; doi:10.3389/fcell.2021.670406)
Supplement: Supplementary file 1 [file Data_Sheet_1.docx]

Table S1 The list of qPCR primers

| No. | Gene | Primer Sequence (5'→3') |
| --- | --- | --- |
| 1 | COX2 | F: 5' TCATAGCAACAAAAGAGTCCACAAA 3' |
|  |  | R: 5' GCCTGCCAAAGTCACAAGCC 3' |
| 2 | PGE2 | F: 5' TGCAGGATGATGTACGGGTAGA 3' |
|  |  | R: 5' AGGCTGGATGTGTGAGTGTCG3' |
| 3 | IL-1β | F: 5' CCAGGACATGCTAGGGAGC3' |
|  |  | R: 5' CAGAGGCAGGGAGGGAAA 3' |
| 4 | IL-6 | F: 5' AAGCCAGAGTCATTCAGAGCAA 3' |
|  |  | R: 5' GCATTGGAAGTTGGGGTAGGA 3' |
| 5 | TNF-α | F: 5' AAAACTCGAGTGACAAGCCCGT 3' |
|  |  | R: 5' TCCCTTGAAGAGAACCTGGGAG 3' |
| 6 | VEGFA | F: 5' AGTATATCTTCAAGCCGTCCTG 3' |
|  |  | R: 5' CATGATCTGCATAGTGACGTTG3' |
| 7 | β-actin | F: 5' GGGAAATCGTGCGTGACATT 3' |
|  |  | R: 5' TGCCCAGGAAGGAAGGCT 3' |
| 8 | VEGFA-P1 | F: 5' GGTTGCTCCTTCACTCCCTC 3'  R: 5' CGCGACTGGTCCGATGA 3' |
| 9 | VEGFA-P2 | F: 5' AAGCCTGCGGTGCTCAAC 3'  R: 5' AGCTGGCAAGGACGTATGG 3' |
| 10 | PDGFB-P1 | F: 5' GCTGTCTCCACCCACCTCTT 3'  R: 5' GTGCGGGTCAGTCTGTCTATCT 3' |
| 11 | PDGFB-P2 | F: 5' TCGGAGACCCTGTCAAGCA 3'  R: 5' AGAAGTTGCCACCCTTCAGC 3' |

Table S2 The list of siRNAs.

| No. | Gene | Primer Sequence (5'→3') |
| --- | --- | --- |
| 1 | COX2-siRNA1 | F: 5' AUCGGGAGUUGGAAUCACUTT 3' |
|  |  | R: 5' AGUGAUUCCAACUCCCGAUTT 3' |
| 2 | COX2-siRNA2 | F: 5' GCCUACUACAAGUGUUUCUTT 3' |
|  |  | R: 5' AGAAACACUUGUAGUAGGCTT3' |
| 3 | VEGFR2-siRNA1 | F: 5' UCCCUGUGAAGUAUCUCAGTT 3' |
|  |  | R: 5' CUGAGAUACUUCACAGGGATT 3' |
| 4 | VEGFR2-siRNA2 | F: 5' ACAUGCUGUGGCACAUAUATT 3' |
|  |  | R: 5' UAUAUGUGCCACAGCAUGUTT 3' |
| 5 | PDGFRβ-siRNA1 | F: 5' GCAGGUGUCAUCCAUCAAUTT 3'  R: 5' AUUGAUGGAUGACACCUGCTT 3' |
| 6 | PDGFRβ-siRNA2 | F: 5' GGUGGUGUUUGAGGCUUAUTT 3'  R: 5' AUAAGCCUCAAACACCACCTT 3' |
| 7 | siCON | F: 5' UUCUCCGAACGUGUCACGUTT 3' |
|  |  | R: 5' ACGUGACACGUUCGGAGAATT 3' |
